# Assessing the Biological Safety of Atmospheric Cold Plasma Treated Wheat Using Cell and Insect Models

**DOI:** 10.3390/foods9070898

**Published:** 2020-07-08

**Authors:** Agata Los, Dana Ziuzina, Robin Van Cleynenbreugel, Daniela Boehm, Paula Bourke

**Affiliations:** 1 Environmental Sustainability and Health Institute, Technological University Dublin, Dublin 7, Ireland; agata.los@tudublin.ie (A.L.); Dana.ziuzina@tudublin.ie (D.Z.); vc.robin@hotmail.com (R.V.C.); daniela.boehm@tudublin.ie (D.B.); 2 Faculty of Engineering Technology, Katholieke University Leuven, 9000 Ghent, Belgium; 3 Plasma Research Group, School of Biosystems and Food Engineering, University College Dublin, Dublin 4, Ireland; 4 School of Biological Sciences, Institute for Global Food Security, Queens University Belfast, Belfast BT9 5DL, UK

**Keywords:** atmospheric cold plasma, food processing, wheat grains, safety evaluations, food model systems, cytotoxicity, mammalian cell line CHO-K1, insect model *Tribolium castaneum*

## Abstract

Atmospheric cold plasma (ACP) is under investigation for an extensive range of biocontrol applications in food biosystems. However, the development of a novel intervention technology requires a thorough evaluation of the potential for negative effects and the implications for the human and animal food chains’ safety. The evaluations were performed using a contained, high-voltage, dielectric barrier discharge plasma system. The cytotoxicity of two types of food models—a liquid model (wheat model medium (WMM)) vs. a solid model (wheat grain extract (WGE)) was compared in vitro using the mammalian cell line CHO-K1. The residual toxicity of ACP treatment of grains for food purposes was assessed using the invertebrate model *Tribolium castaneum*, by feeding the beetles with flour produced from ACP-treated wheat grains. The cytotoxic effects and changes in the chemistry of the ACP-treated samples were more pronounced in samples treated in a liquid form as opposed to actual wheat grains. The feeding trial using *T. castaneum* demonstrated no negative impacts on the survivability or weight profiles of insects. Investigations into the interactions of plasma-generated species with secondary metabolites in the food matrices are necessary to ensure the safety of plasma for food applications.

## 1. Introduction

The disruptive potential of cold plasma for the safety and quality enhancement of a wide range of food products, with an emerging focus on the earlier stages of food production chains including agricultural pre-treatments for seeds, water or soil, is under increased investigation. However, attention is required on the effects on food components integrity, potential for knock-on toxicity and the safety of this emerging technology for application to different food commodities. Research performed in the context of biomedical applications of plasma has demonstrated the cytotoxic properties of plasma-treated solutions (both water and more complex media, containing carbohydrates, lipids and/or proteins) in mammalian cell models [1,2]. The cytotoxic and mutagenic effects of plasma-treated, protein-based solutions and produce-based solutions have also been reported [3,4,5]. For instance, water, phosphate-buffered saline and phosphate buffer solution exposed to plasma for 10 min resulted in cell death in more than 50% of cells [1]. Foetal bovine serum treated with plasma for 5 or 10 min caused not only strong growth inhibition of HeLa cells, but also exhibited increased mutagenic potential [3].

Chemical reactions between food components and plasma reactive species are responsible for food surface modifications and functionality changes, however, knowledge of the direct interactions between plasma-generated ROS/RNS and components, such as carbohydrates, complex lipids, and proteins, is limited, especially regarding the impact of potentially formed toxic products [6,7]. There is a need to evaluate the safety of a wide range of ACP-treated food matrices in order to demonstrate that their consumption is non-hazardous and for regulatory considerations.

The objective of this study was to evaluate the safety profile of ACP-treated cereal grain models using the well-established mammalian cell model CHO-K1. The cytotoxicity of two types of plasma-exposed wheat models was determined, including a liquid model (wheat model medium (WMM)) and a solid model (wheat grain extract, WGE-prepared using wheat grains exposed to plasma). Additionally, the safety of using cold plasma for grains processing and ingestion, i.e., the residual toxicity of plasma treatment, was assessed using an insect feeding trial using the invertebrate model *Tribolium castaneum*. The beetles were fed with flour produced from plasma-treated grains.

## 2. Materials and Methods

### 2.1. Wheat Grains

Organic wheat (origin: United Kingdom) grains were purchased from a local supermarket.

### 2.2. Preparation of Wheat Model Medium (WMM)

Wheat grains (10 g) were milled using a Nutribullet blender (model NB-101B) for 10 s to obtain fine and homogeneous flour. Wheat model medium (WMM) was prepared as for the method described by Charalampopoulos et al. [8] with minor modifications. The resulting wheat flour (50 g) was mixed with 450 mL of tap water and centrifuged at 6000 *g* for 30 min at room temperature. The supernatant was sterilized at 121 °C for 45 min. The procedures of centrifugation and sterilization were repeated four times to minimise the presence of sediments caused by sterilization in the final media. Prepared wheat model media were stored at 4 °C before use. Three independent ACP treatment experiments were conducted.

### 2.3. ACP System Set Up

**Figure 1:**
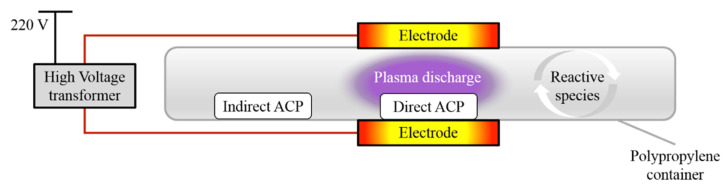
Dielectric barrier discharge Atmospheric Cold Plasma generator set-up. Adapted from Ziuzina et al. [10]

The ACP system used in this study was a dielectric barrier discharge system with a maximum high voltage output of 120 kV at 50 Hz. The system has been previously described by [9] and a schematic diagram is presented in Figure 1. The distance between the two aluminium electrodes was equal to the height of the polypropylene container (20 mm), used as a sample holder and a dielectric barrier. For direct treatment, the distance between the top electrode and the sample was approximately 15 mm. For indirect treatment, the distance between the samples and centre of the electrodes varied from 120 to 160 mm owing to the sample distribution in the petri dish.

The plasma working gas was atmospheric air.

### 2.4. Plasma Treatment

Samples of wheat grains (10 g, evenly distributed single layer of grains, with thickness equal to the thickness of single grain ~2–3 mm) or WMM (10 mL, evenly distributed) in petri dish were exposed to 80 kV_RMS_ ACP for 5 or 20 min with both direct and indirect plasma treatments conducted simultaneously. For direct exposure, samples were placed between the electrodes, i.e., within the plasma discharge, while, for indirect plasma treatment, samples were placed in the corner of the container, i.e., outside plasma discharge (Figure 1). Before treatment, each container was sealed with a high-barrier polypropylene bag (catalog number B2630; Cryovac, Dublin, Ireland). After treatment, the samples were stored at 15 °C for 24 h. Three independent ACP treatment experiments were conducted.

### 2.5. Preparation of WGE

The ACP-treated wheat grains (10 g) were milled using a Nutribullet blender for 10 s to obtain fine and homogeneous flour. This flour (2.2 g) was mixed with tap water (20 mL) (proportions as per WMM preparation procedure, Section 2.2.), shaken for 60 min at 200 rpm and centrifuged at 6000× *g* for 30 min. The supernatant, i.e., wheat grain extract (WGE), was filter-sterilised and used for cytotoxicity and further analyses.

Untreated and ACP-treated wheat model medium (WMM) and wheat grain extract (WGE) prepared using either untreated or ACP-treated wheat grains, were used for safety evaluations.

### 2.6. Cytotoxicity Assay

The cytotoxicity assay was performed according to the protocol by Boehm et al. [11] using Chinese hamster ovary (CHO-K1) cell line obtained from culture stocks of TU Dublin or University College Dublin. Cytotoxicity of the treated medium was measured by assessing adherent cell mass using crystal violet assay. Cells were cultured in Dulbecco’s modified Eagle’s medium/Ham’s F12 Nutrient Mixture (DMEM/F12) supplemented with 2 mM L-glutamine and 10% foetal bovine serum (FBS), supplemented with 2%, 5% and 10% of either WMM or WGE (*v*/*v*). CHO-K1 cells were grown at 37 °C and 5% CO_2_ in a humidified incubator. The growth of cells seeded at 2.5 × 10^4^ cells/mL after 3 days of culture was assessed by crystal violet staining according to the protocol described by Tsoukou et al. [1], with minor modifications. Briefly, the culture supernatant was removed and attached cells were fixed with methanol (70%) for 1 min and followed by staining with crystal violet solution (0.2%) for 10 min. Excess stain was rinsed off, plates were air-dried, and the crystal violet bound to the adherent cells was re-solubilized using acetic acid (10%). The absorbance was measured at 560 nm on a spectrophotometric microplate reader (BioTek, Winooski, VT, USA). Cell growth was expressed as a percentage of control cells grown in medium supplemented with untreated WMM or WGE. To assess the effect of untreated WMM or WGE on CHO-K1 cells, cell growth was also compared to cells grown in medium supplemented with sterile, untreated PBS. As stated above, three independent ACP treatment experiments were conducted, and for each condition the sample absorbance was read in triplicate (three individual wells of the 96-well plate), resulting in total number of nine per condition to be evaluated (*n* = 9).

### 2.7. Chemical Properties of WMM and WGE

Hydrogen peroxide (H_2_O_2_) concentration measurement was based on the oxidation of potassium iodide (KI; Sigma Aldrich, Arklow, Ireland) to iodine and spectrophotometric measurement at 390 nm [3]. Briefly, 50 μL of phosphate buffer and 100 μL of 1 M KI solution were added to 50 μL of sample. After 30 min incubation, absorbance was read on a spectrophotometric plate reader at 390 nm. Each sample (of three independent experiments) absorbance was read in triplicate (three individual wells), resulting in total number of nine per each condition to be evaluated (*n* = 9).

The concentration of nitrite (NO_2_^−^) was determined using Griess reagent (Sigma Aldrich, Ireland) according to the procedure described by Lu et al. [12]. For 100 μL of sample, 100 μL of Griess reagent was added, and absorbance was read at 548 nm after 30 min incubation. Each sample (of three independent experiments) absorbance was read in triplicate (three individual wells) resulting in total number of nine per each condition to be evaluated (*n* = 9).

The concentration of nitrate (NO_3_^−^) was determined photometrically by 2,6-dimethyl phenol (DMP) using the Spectroquant nitrate assay kit (Merck, Germany). Samples were pre-treated with sulfamic acid to eliminate nitrite interference. Each sample’s (of three independent experiments) absorbance was read in duplicate (two individual wells), resulting in a total number of six per each condition to be evaluated (*n* = 6).

A standard curve of known H_2_O_2_ ((Perhydrol^®^) for analysis EMSURE^®^ ISO), NO_2_^−^ (sodium nitrite, Sigma Aldrich, Arklow, Ireland) and NO_3_^−^ (sodium nitrate, Sigma Aldrich, Arklow, Ireland) concentrations was included on each assay plate and used to convert absorbance into the concentration values. Each sample’s (of three independent experiments) absorbance was read in duplicate (two individual wells), resulting in total number of six per each condition to be evaluated (*n* = 6).

The pH was determined by potentiometric measurement of the suspensions at 25 °C using an Orion pH meter (model 420A). As stated above, three independent ACP treatment experiments were conducted and, for each condition, the sample’s pH was measured once (*n* = 3).

### 2.8. Optical Microscopy

Microscopic analysis of cells was performed using inverted light microscope (Optika, Pontarenica, Italy, Tucsen Photonics, Co., Ltd.) and a total magnification of 200 X.

### 2.9. Insect Feeding Trial

Culture rearing conditions: *Tribolium castaneum* obtained from Blades-Biological (Kent, UK) was reared in plastic containers on 95% organic wheat flour supplemented with 5% brewer’s yeast mixture at 34 °C in the dark. Whole wheat grains (10 g) exposed to direct and indirect plasma treatment for 20 min and post-treatment storage for 24 h at 15 °C were used to prepare flour as food for insects. Three independent ACP treatment experiments were conducted. Control flour feed was produced from grains, which were untreated and stored for 24 h at 15 °C. Immediately after the treatment and post-treatment storage, each sample was ground for 10 s, five times to obtain fine particles using a Nutribullet blender. The resultant flour was sieved to produce particle size of <800 µm, which was then used as the rearing medium for insects. Each sample was divided in two equal aliquots of ~4.5 g resulting in six samples to be evaluated per each condition (*n* = 6). A 4.5 g aliquot of each sample (either ACP-treated or untreated control) was used to feed *T. castaneum*-second instar larvae (2–3 days old or 7 days from oviposition, 25 larvae per sample). The samples were incubated at 34 °C in the dark until the emergence of adults in all samples was observed (5 weeks from the time of ACP treatment and inoculation). The mortality of insects was calculated as the number of dead insects/total number of insects for each trial × 100. The weight of adult insects (measured in a pool of 20 insects) for each experimental group was recorded. Each experiment was performed in duplicate and repeated three times (*n* = 6).

The magnitude of lipid peroxidation, a non-enzymatic oxidative stress marker, was determined by measuring concentration of malondialdehyde (MDA, a by-product of lipid peroxidation, which is thiobarbituric acid reactive substance) according to the procedure described by Wang et al. [13] with minor modifications. Briefly, twenty living adult insects were homogenized by crushing the beetles with 1 mL of ice-cold phosphate-buffered saline (PBS). The homogenates were centrifuged twice for 10 min at 10,000 rpm. To 0.2 mL of supernatant 0.2 mL of 30% tri-chloroacetic acid (Sigma-Aldrich, Arklow, Ireland) was added. The mixture was vortexed and centrifuged at 10,000 rpm for 10 min at 4 °C. After centrifugation, 0.3 mL of supernatant was mixed with 0.3 mL of 0.8% thiobarbituric acid (Sigma-Aldrich, Arklow, Ireland). The resulting suspension was incubated at 98 °C for 60 min, cooled on ice for 5 min and centrifuged at 4000 *g* for 1 min. The concentration of MDA in test solutions was determined by measuring absorbance in duplicate (two individual wells per sample) at 532 nm, and comparing to a standard curve of known MDA (Sigma-Aldrich, Arklow, Ireland) concentrations. The MDA level was expressed as μmol per g of weight of insects (*n* = 6).

### 2.10. Statistical Analysis

Statistical analysis was performed using IBM SPSS statistics 21 Software (SPSS Inc., Chicago, IL, USA). Significant differences between samples were determined using one-way analysis of variance (ANOVA), and Fisher’s Least Significant Difference (LSD) at the 0.05 level.

## 3. Results

### 3.1. The Effect of ACP-Treated WMM and WGE on Mammalian Cell Growth

**Figure 2:**
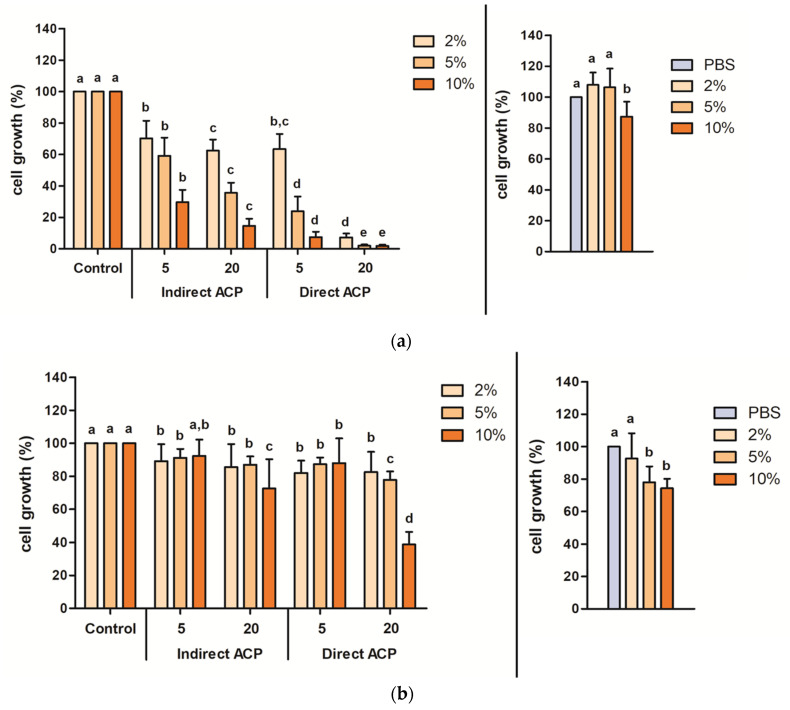
Cell growth of CHO-K1 in response to supplementation with (**a**) WMM and (**b**) WGE determined in cytotoxicity assay. Cell growth (%) assessed by CV assay, shown as function of the respective control supplemented with untreated WMM/WGE (left panel) or cells supplemented with PBS (right panel). Left panel: cell growth was expressed as a percentage of control cells grown in medium supplemented with untreated WMM or WGE. Different letters indicate significant difference between samples supplemented with untreated and ACP-treated WMM/WGE at the same supplementation level (left panel) or between samples supplemented with PBS and untreated WMM/WGE (right panel) (*p* < 0.05). Results presented as mean absorbance values of three independent experiments recorded in triplicate (*n* = 9). Vertical bars represent standard deviation.

The effect of WMM and WGE on the growth of CHO-K1 cells was assessed by supplementing the cell culture medium with these solutions at 2%, 5% and 10% (*v*/*v*) and determining the total adherent cell mass after 3 days of culture by crystal violet staining. When the cell culture media was supplemented with untreated WMM at 2 or 5% (*v*/*v*), it stimulated cell growth slightly by 7.9% and 6.4%, respectively (no significant difference in respect to media supplemented with PBS). Supplementation with 10% untreated WMM had a negative effect on cell growth—a significant decrease by 12.6% was observed (Figure 2a, right panel). The negative effect of supplementation with untreated WGE on cell growth was more pronounced for all % levels, where 5% and 10% (*v*/*v*) levels significantly decreased cell growth by 22.0% and 25.7%, respectively (Figure 2b, right panel).

The reduction in cell growth was dependent on the type of the solution tested (WMM vs. WGE) as well as plasma treatment parameters (Figure 2, left panels). In general, a longer plasma treatment time (20 min as compared to 5 min), direct exposure (as opposed to indirect) and the highest supplementation level tested (10%) showed the most toxic effect on cells for both WMM and WGE. It was clear that plasma-treated WMM had a more toxic effect on cells than WGE, with decreases in cell growth by an average 96.3 % for supplementation at all levels (2–10%), respectively (20-min direct treatment) (Figure 2a, left panel). For the same treatment parameters, i.e., 20-min direct plasma exposure, WGE decreased cell growth by 17.5%, 22.2%, and 61.2% for supplementation of 2%, 5% and 10%, respectively (Figure 2b, left panel). It should be noted that for WGE samples, supplementation at 10% with 20-min exposure only (both direct and indirect) resulted in a decrease in cell growth higher than 25%, whereas for WMM, such decreases were noted for all samples and for all plasma treatment, durations and modes of exposure.

**Figure 3:**
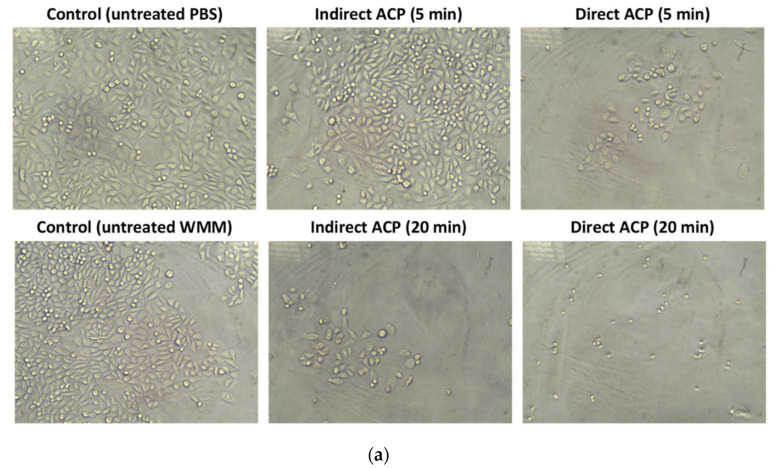

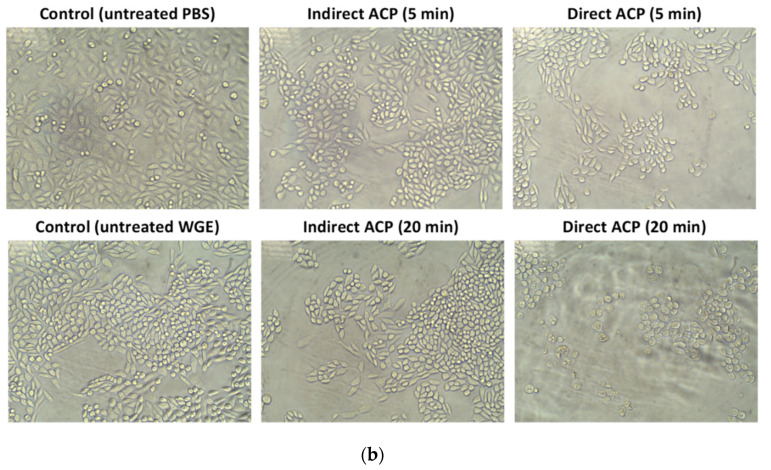
Morphology of the CHO-K1 cells incubated in medium supplemented with (**a**) WMM and (**b**) WGE. Magnification: 200x. Supplementation level: 10% (*v*/*v*). ACP treatment parameters: treatment time—5 and 20 min; post-treatment storage time—24 h.

The results of the cytotoxicity assay were in line with observations of cellular morphology changes of CHO-K1 cells after incubation in medium supplemented with either WMM or WGE—the most pronounced differences as compared to the control were observed for the samples for which the cytotoxic effect was the strongest, i.e., 20-min direct plasma treatment. Light microscopy analyses revealed that the cells incubated in medium supplemented with the plasma-treated liquids exhibited striking changes in morphology and cell growth compared to control cells—they failed to develop a regular cell morphology and displayed increased round cell morphology compared to the normal epithelial cell shape. Moreover, the number of cells attached to the bottom of the plate was reduced after cultivation with plasma-treated liquids (Figure 3).

### 3.2. Chemical Properties of WMM and WGE

**Figure 4:**
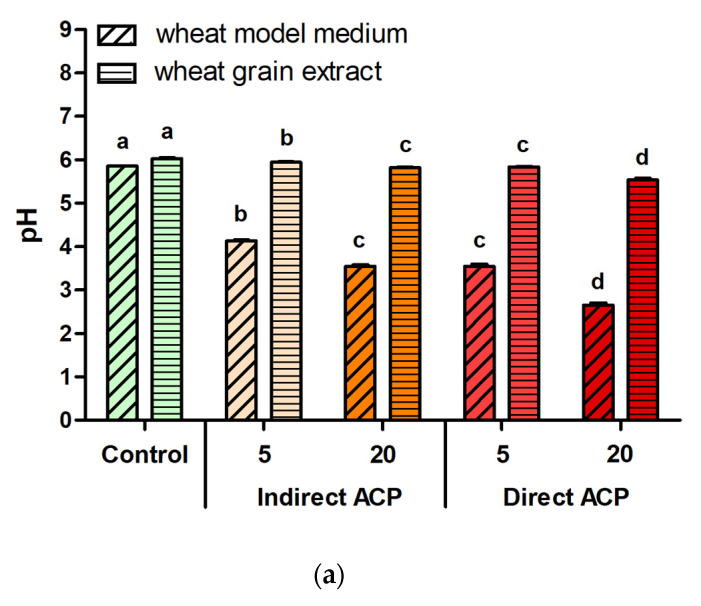

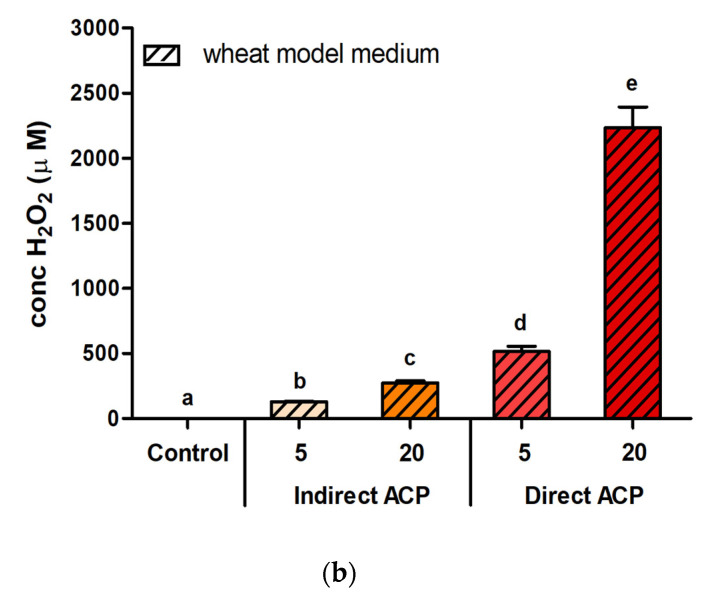

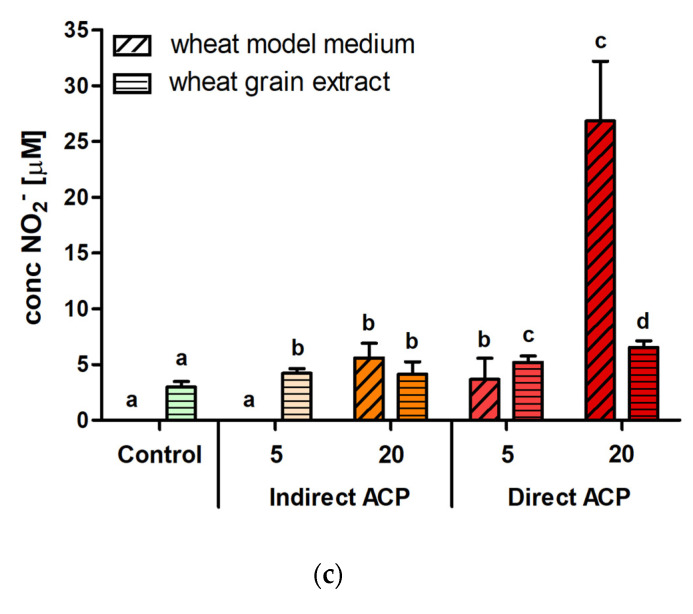

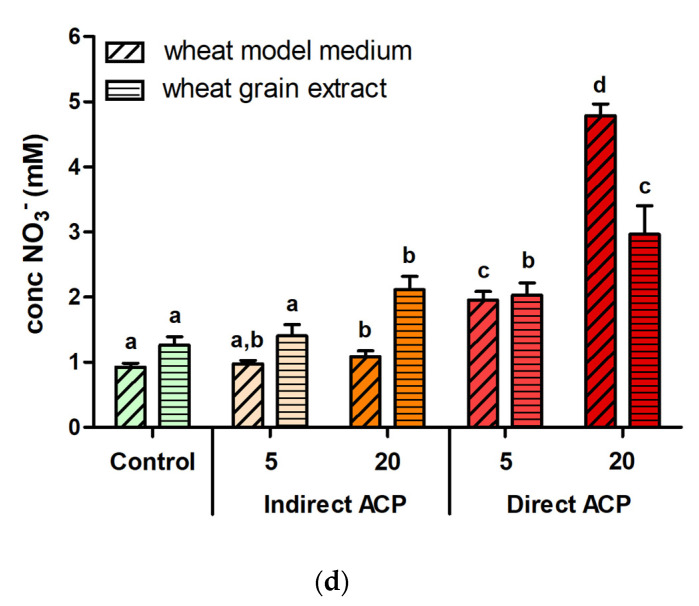
Effect of plasma treatment on chemical properties of WMM and WGE: (**a**) pH values, concentrations of (**b**) hydrogen peroxide, (**c**) nitrites and (**d**) nitrates. Different letters indicate significant difference between untreated control and ACP-treated samples within the same sample type (*p* < 0.05). Results presented as mean absorbance values of three independent experiments. Vertical bars represent standard deviation.

As exposure to ACP can result in the generation of high concentrations of compounds that can mediate the cytotoxic effects on mammalian cells [11], it is necessary to monitor the effect of plasma treatment on chemical composition and potential changes occurring within a liquid medium and solid substrates. Changes in the pH values of the liquid media, as well as the change in the concentrations of hydrogen peroxide (H_2_O_2_), nitrites (NO_2_^−^) and nitrate (NO_3_^−^) in untreated and plasma-treated samples were monitored (Figure 4).

The pH levels were reduced, with the most pronounced decrease noted for 20-min direct treatment, which resulted in a pH reduction from 6.0 (untreated control) to 5.5 for WGE, and a more dramatic decrease from 5.8 (untreated control) to 2.7 for WMM (Figure 4a). H_2_O_2_ was detected in ACP-treated WMM but not in WGE samples, and no H_2_O_2_ was observed in untreated samples. The maximal value of H_2_O_2_ (2234.0 µM) was measured in directly treated 20-min WMM (Figure 4b). NO_2_^−^ was not present in untreated WMM, however, very low concentrations were detected post plasma treatment, with concentrations dependent on the mode of plasma exposure. Higher concentrations of NO_2_^−^ were reported for samples treated directly (5.6 and 26.8 µM for treatment times of either 5 or 20 min, respectively) than indirectly (3.7 µM for 20-min treatment; no NO_2_^−^ was detected for 5-minute exposure). In the case of WGE samples, low concentrations of NO_2_^−^ were detected in both untreated and ACP-treated samples. Overall, the increase of NO_2_^−^ in WGE samples was lower than for WMM—concentrations of NO_2_^−^ increased from a baseline of 3.0 µM present in untreated WGE controls to 6.5 µM after 20-min direct treatment (the highest concentration of NO_2_^−^ among all tested parameters for WGE) (Figure 4c). In contrast, NO_3_^−^ was present in both untreated WMM and WGE samples. The concentrations of NO_3_^−^ increased after ACP treatment, reaching maximal values after 20-min direct plasma exposure, increasing from 0.9 mM to 4.8 mM (20-min direct ACP) for WMM and from 1.3 mM to 3.0 mM (20-min direct ACP) for WGE. The highest concentration of NO_3_^−^ was detected in WMM treated with direct plasma for 20 min (Figure 4d).

### 3.3. Cell Growth in the Presence of H_2_O_2_

**Figure 5:**
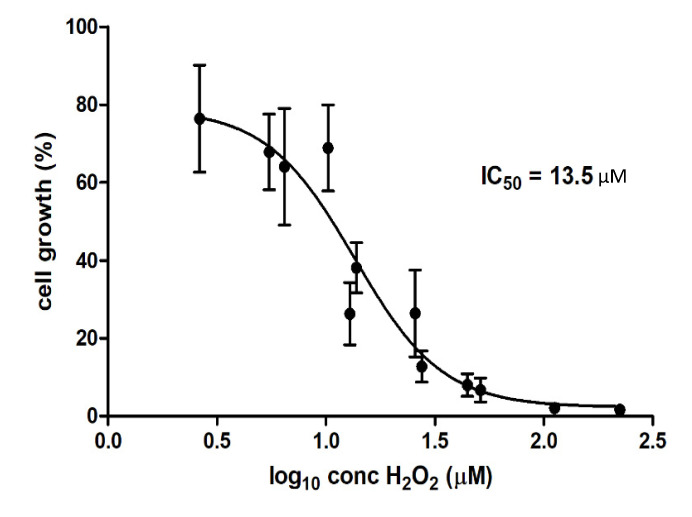
Dose–response curve of CHO-K1 cells to hydrogen peroxide detected in WMM. Vertical bars represent standard deviation.

Hydrogen peroxide has been identified as one of the principle mediators of cytotoxic effects in plasma-treated liquids on mammalian cells, and cell death tends to correlate with the hydrogen peroxide concentrations of the solution [14]. Cell growth (%) in response to supplementation with WMM (2, 5 and 10%) was plotted as a function of the concentration of H_2_O_2_ detected in WMM samples and fitted with a response-inhibition curve to obtain an IC50 of 13.5 µM (Figure 5). The H_2_O_2_ concentrations presented in Figure 5 were calculated from the concentrations measured in the treated WMM and the respective dilutions into the cell culture medium at 2, 5 and 10% (*v*/*v*) in order to explore the common effect of H_2_O_2_ across all supplementation conditions.

### 3.4. Insects Studies

**Figure 6:**
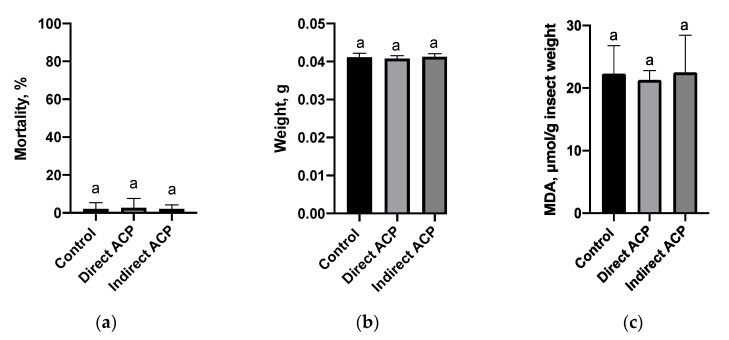
Mortality (**a**), weight (**b**) and MDA content (**c**) for *T. castaneum* reared on flour produced from grains exposed to direct and indirect 20 min ACP treatment. Different letters indicate significant difference between untreated control and ACP-treated samples within the same sample type (*p* < 0.05). Results presented as mean absorbance values of three independent experiments conducted in duplicate (*n* = 6). Vertical bars represent standard deviation.

Flour that was prepared from ACP-treated (20 min) grains and subsequently used as the food source to maintain the *T. castaneum* did not significantly affect insects’ viability markers. The mortality of insects reared on flour produced from control, directly and indirectly treated grains was 2.0 (±3.4), 2.6 (±4.8) and 2.0 (±2.2) %, respectively (Figure 6a). The recorded weight of insects (Figure 6b) was 0.0411 (±0.0009) g for control sample and 0.0408 (±0.0007) and 0.0412 (±0.0008) g for directly and indirectly treated wheat grains, respectively.

There was no increase in the MDA content recorded for the insects fed by flour produced from plasma-treated grains, suggesting that no lipid peroxidation, as a response to stress, occurred. The concentrations recorded for control, direct and indirect modes of plasma treatment corresponded to 24.03 ± 1.67, 21.31 ± 1.50 and 24.49 ± 3.95 μM per g of insect weigh, respectively (Figure 6c).

## 4. Discussion

Assessing the potential for induced modifications or toxicity of ACP-treated food matrices is essential to ensure safe process development and general application in the food industry.

For untreated cereal models, supplementation with the highest tested concentration, i.e., 10% (*v*/*v*), of WMM and WGE resulted in a significant decrease in CHO-K1 cell growth by 12.6% for WMM and 25.7% for WGE. In a safety evaluation of a fresh produce model—a lettuce broth—the cell growth of CHO-K1 cells was unaffected when supplementing the cell culture media with up to 10% *v*/*v* of undiluted, untreated lettuce broth, showing that, unlike the grain samples, the lettuce broth itself did not adversely affect the cells [5]. The growth reduction caused by untreated WMM and WGE could be the result of certain grain components having growth inhibitory effects or interfering with the cells’ adhesion to the culture plate surface. The negative effect of WMM and WGE on the cells’ growth could also suggest that the approach used in this study may overestimate the toxicity for certain food models. Allegra et al. [15] stated that cell culture systems may overestimate the toxicity of some chemicals, especially low-toxicity chemicals, and suggested the use of insect models, such as *Galleria mellonella* (wax moth) as a potential alternative, which, in their study was found to be a reliable predictor for low toxicity chemicals.

The cytotoxicity and changes in chemistry of the ACP-treated samples were hugely dependent on the sample type (liquid vs. solid) and the effects were more pronounced in samples treated in a liquid form. Visually observable changes were found immediately after ACP treatment in WMM samples (change in colour from yellowish to transparent), whereas no change in the colour was noted in WGE (Figure S1). ACP-treated WMM (treated as a liquid) had a much stronger toxic effect on cells than WGE (treated as solid wheat grains). WMM and WGE could differ in the composition and probably also in the concentration of major components such as protein and carbohydrates due to the difference in the media preparation where thermal and centrifugation steps were not included for the preparation of WGE. The toxic effect of WMM and WGE was affected by both supplementation levels in the cell culture media and ACP treatment parameters, exhibiting the highest decrease in cells’ growth at 10% supplementation with samples subjected to 20-min direct plasma exposure for both WMM (decrease by 98.2%) and WGE (decrease by 61.2%). Using the same DBD–ACP setup, Heslin et al. [5] evaluated the safety of ACP-treated lettuce broth using in vitro and in vivo toxicity models-CHO-K1 cells and *Galleria mellonella* larvae, respectively. A discrepancy between the results for mammalian cell model and larvae were reported. After 1 min of ACP treatment, the cell growth of CHO-K1 cells in the presence of 10% *v*/*v* plasma-treated lettuce broth decreased to 74% compared to the untreated control. After the initial decrease, cell growth remained stable at 68% compared to the untreated control when supplemented with lettuce broth ACP-treated for up to 10 min. Although low cytotoxic effects were detected in vitro, a strong response of the *Galleria* larvae (in vivo model) to injection with plasma-treated lettuce broth was observed for 5-minute treated broth, with less than 10% larvae survival. These results indicate the importance of assessing ACP-treated products in multiple toxicity models and, ultimately, in the context of ingestion systems. The higher toxicity in vitro of cereal-based models used in this study as compared to lettuce broth could be attributed to the different medium complexity and composition [16] with potential for both carbohydrate, lipid and protein fractions of the wheat flours to undergo structural changes as a result of degradation or oxidation [17,18]. ACP-treated WMM and WGE resulted in cytotoxic effects on mammalian cells. Plasma treatment of aqueous solutions generates reactive species in the liquid, including hydrogen peroxide, nitrates, and nitrites, which may react to form further cell toxic compounds, such as peroxynitrite/peroxynitrous acid [11]. Hydrogen peroxide (H_2_O_2_) can induce DNA damage, cause cell cycle arrest and trigger apoptosis. Its toxicity is determined largely by the cellular anti-oxidant status and ability to detoxify H_2_O_2_ through catalase [11]. Although there was a plasma treatment time-dependent increase in H_2_O_2_ in lettuce broth, after 10-minute treatment it was still less than 80 µM, potentially due to high concentrations of antioxidants such as vitamins and polyphenols in the lettuce [5], whereas the values of H_2_O_2_ in WMM reached levels approximately 25× higher (2234.0 µM after 20-min direct treatment). Peroxide levels in treated PBS (buffer without organic molecules) were determined to be in the range of 200–400 μM with a treatment duration of 5–10 min, with nitrite and nitrate concentrations around 50 µM and 500 µM, respectively [1,3].

Hydrogen peroxide, however, was not the only cytotoxic factor of the tested cereal models. There was no H_2_O_2_ detected in ACP-treated WGE samples, yet they also showed cytotoxic effects, which could be possibly attributed to other changes observed in chemical composition post-plasma treatment, i.e., decrease in pH and increase in nitrites and nitrates. However, as pointed out by Boehm et al. [11], the addition of plasma-activated water (PAW) up to concentrations of a total of 80% (*v*/*v*) did not result in an acidification of DMEM/F12 medium, which is buffered both with HEPES and bicarbonate and contains 10% FBS, and therefore the pH-related effects of PAW and other plasma-treated solutions when diluted in DMEM can be discounted. While medium components such as pyruvate or BSA may also act as scavengers of H_2_O_2_, this would further reduce the concentrations of H_2_O_2_ cells were exposed to and support a role for other cytotoxic factors. The final concentrations of nitrite and nitrate resulting from supplementation with WMM did not exceed 5 µM and 0.5 mM, respectively, while up to 1.2 mM nitrite or nitrate were well-tolerated in the same cell model [11]. In addition to these effects, significant increases in the concentrations of malondialdehyde (MDA) were also observed for ACP-treated wheat grains [18]. MDA, a product of lipid peroxidation, is known for its cytotoxic properties. MDA is able to form adducts with proteins that lead to their inactivation [19], as well as react with DNA to form adducts with deoxyguanosine and deoxyadenosine [20].

In this study, the IC50 of 13.5 µM for H_2_O_2_ was obtained for WMM samples (H_2_O_2_ was not present in WGE). Boehm et al. [11] reported that the IC50 values of H_2_O_2_ for CHO-K1 cells for PAW were much lower than those obtained through supplementation of pure H_2_O_2_ into the cell medium, obtaining IC50 of 105 µM and 225 µM for PAW and pure H_2_O_2_, respectively. IC50 obtained for WMM was therefore almost 10-fold lower than for PAW and more than 10-fold lower than for pure H_2_O_2_, confirming that although cytotoxic properties correlate with H_2_O_2_ concentrations in plasma-treated solutions, other factors contributing to plasma-induced cytotoxicity are involved, and may act synergistically with H_2_O_2_.

Beetles are the largest insect order, with their representative, *Tribolium castaneum* Herbst (Coleoptera: Tenebrionidae) the red flour beetle, emerging as a new potential candidate for a biological model organism and bio-indicator in environmental and biomedical studies due to the similarity of their signalling pathways, basic immune mechanisms, energy metabolism and structural components with mammals [21]. *T. castaneum* is also a global pest of stored food products including wheat grains [22], therefore it is widely employed in studies testing novel insecticides of physical, chemical and biological origin [23,24,25]. This study evaluated the residual toxicity of plasma treatment as a potential method for biological control of stored grains using *T. castaneum* as a model organism. In contrast to the in vitro cytotoxicity assay, flour produced from plasma-treated grains did not affect the viability and weight of insects regardless of the mode of plasma exposure. This can be due to the lower sensitivity of *Tribolium* as a model to detect observed chemical fluctuations in flour. It is known that in insects under the influence of environmental and physiological stressors, an enhanced production of ROS can occur. In the cell membrane, polyunsaturated fatty acids are the primary target for ROS stimulating the process of lipid peroxidation and MDA, as one of the secondary products of lipid peroxidation, is used to evaluate the extent of oxidative stress in insects [26,27]. Lipid peroxidation level, measured in insects fed on flour generated from directly treated grains, was slightly lower than in the control population or remained unchanged, as noted in insects fed on the flour of indirectly treated grains, suggesting that antioxidant processes were sufficient, or an absence of oxidative stress, and thus grain toxicity to insects after plasma exposure. It is also possible that any lipid peroxidation would occur during the first hours, which is later repaired over the 5 weeks of incubation. A slight reduction in the MDA content could also be associated with antioxidant responses and the change in the level of proteins or peptides known to decrease oxidative stress in insects. For example, caffeine supplementation of food markedly increased total thiol and non-protein thiol glutathione levels in cockroaches, which was associated with significant reduction of lipid peroxidation [28]. In contrast, Holmstrup et al. [29] observed a decrease in lipid peroxidation in *T. castanaeum*, which was related to oxidative stress, indicating the general depression of insects’ metabolism. Although insects’ feeding, as a part of the preliminary “warning” study, indicated no negative impacts, further investigations are warranted to examine the immediate effect of plasma-treated components on MDA content and to study the molecular and physiological responses, enzymes and non-enzymatic systems in this and other model organisms to understand if ACP treatment mediates toxic effects to ensure safe application of this technology across agricultural and food sectors.

## 5. Conclusions

The cytotoxic effects and changes in the chemistry of the ACP-treated samples were much more pronounced in samples treated in a liquid model form as opposed to actual wheat grains. The exposure of both types of cereal models to ACP resulted in a pH decrease, as well as an increase in nitrate and nitrite concentrations. High concentrations of hydrogen peroxide were observed in ACP-treated WMM after extended direct treatment (20 min), but an IC50 value of 13.5 µM, which is more than an order of magnitude lower than the cells’ tolerance, suggested a strong relevance for other toxic components. Investigations of the interactions of plasma-generated species with secondary metabolites in the food matrices are necessary to ensure the safety of plasma for food applications. This study, evaluating plasma toxicity using a *T. castaneum* feeding trial, demonstrated no negative impacts of plasma-treated dry material on the survivability and weight profile of the insects. The demonstrated reduction in lipid peroxidation in the insects reared on plasma-treated flour warrants detailed metabolic investigation.

## Supplementary Materials

The following are available online at https://www.mdpi.com/2304-8158/9/7/898/s1, Figure S1: Change in the color of (**a**) WMM compared to (**b**) WGE after subjecting to ACP treatment.

## References

[1] Tsoukou E., Bourke P., Boehm D. (2018). Understanding the differences between antimicrobial and cytotoxic properties of plasma activated liquids. Plasma Med..

[2] Griseti E., Kolosnjaj-Tabi J., Gibot L., Fourquaux I., Rols M.P., Yousfi M., Merbahi N., Golzio M. (2019). Pulsed electric field treatment enhances the cytotoxicity of plasma-activated liquids in a three-dimensional human colorectal cancer cell model. Sci. Rep..

[3] Boehm D., Heslin C., Cullen P.J., Bourke P. (2016). Cytotoxic and mutagenic potential of solutions exposed to cold atmospheric plasma. Sci. Rep..

[4] Bourke P., Ziuzina D., Han L., Cullen P.J., Gilmore B.F. (2017). Microbiological interactions with cold plasma. J. Appl. Microbiol..

[5] Heslin C., Boehm D., Gilmore B.F., Megaw J., Bourke P. (2020). Safety evaluation of plasma-treated lettuce broth using in vitro and in vivo toxicity models. J. Phys. D Appl. Phys..

[6] Wende K., Schmidt A., Bekeschus S. (2018). Safety aspects of non-thermal plasmas. Comprehensive Clinical Plasma Medicine.

[7] Zhang K., Perussello C.A., Milosavljević V., Cullen P.J., Sun W., Tiwari B.K. (2019). Diagnostics of plasma reactive species and induced chemistry of plasma treated foods. Crit. Rev. Food Sci. Nutr..

[8] Charalampopoulos D., Pandiella S.S., Webb C. (2002). Growth studies of potentially probiotic lactic acid bacteria in cereal-based substrates. J. Appl. Microbiol..

[9] Ziuzina D., Boehm D., Patil S., Cullen P.J., Bourke P. (2015). Cold plasma inactivation of bacterial biofilms and reduction of quorum sensing regulated virulence factors. PLoS ONE.

[10] Ziuzina D., Patil S., Cullen P.J., Keener K.M., Bourke P. (2013). Atmospheric cold plasma inactivation of Escherichia coli in liquid media inside a sealed package. J. Appl. Microbiol..

[11] Boehm D., Curtin J., Cullen P.J., Bourke P. (2018). Hydrogen peroxide and beyond-the potential of high-voltage plasma-activated liquids against cancerous cells. Anticancer Agents Med. Chem..

[12] Lu P., Boehm D., Bourke P., Cullen P.J. (2017). Achieving reactive species specificity within plasma-activated water through selective generation using air spark and glow discharges. Plasma Process. Polym..

[13] Wang L., Cui S., Liu Z., Ping Y., Qiu J., Geng X. (2018). Inhibition of mitochondrial respiration under hypoxia and increased antioxidant activity after reoxygenation of Tribolium castaneum. PLoS ONE.

[14] Winter J., Tresp H., Hammer M.U., Iseni S., Kupsch S. (2014). Tracking plasma generated H_2_O_2_ from gas into liquid phase and revealing its dominant impact on human skin cells. J. Phys. D Appl. Phys..

[15] Allegra E., Titball R.W., Carter J., Champion O.L. (2018). Galleria mellonella larvae allow the discrimination of toxic and non-toxic chemicals. Chemosphere.

[16] Los A., Ziuzina D., Boehm D., Cullen P.J., Bourke P. (2017). The potential of atmospheric air cold plasma for control of bacterial contaminants relevant to cereal grain production. Innov. Food Sci. Emerg. Technol..

[17] Bahrami N., Bayliss D., Chope G., Penson S., Perehinec T., Fisk I.D. (2016). Cold plasma: A new technology to modify wheat flour functionality. Food Chem..

[18] Los A., Ziuzina D., Boehm D., Cullen P.J., Bourke P. (2019). Investigation of mechanisms involved in germination enhancement of wheat (Triticum aestivum) by cold plasma: Effects on seed surface chemistry and characteristics. Plasma Process. Polym..

[19] Argüelles S., Machado A., Ayala A. (2009). Adduct formation of 4-hydroxynonenal and malondialdehyde with elongation factor-2 in vitro and in vivo. Free Radic. Biol. Med..

[20] Gentile F., Arcaro A., Pizzimenti S., Daga M., Paolo Cetrangolo G., Dianzani C., Lepore A., Graf M.R.J., Ames P., Barrera G. (2017). DNA damage by lipid peroxidation products: Implications in cancer, inflammation and autoimmunity. AIMS Genet..

[21] Adamski Z., Bufo S.A., Chowański S., Falabella P., Lubawy J., Marciniak P., Pacholska-Bogalska J., Salvia R., Scrano L., Słocińska M. (2019). Beetles as model organisms in physiological, biomedical and environmental studies - A review. Front. Physiol..

[22] Din N., Ashraf M., Hussain S., Hussain D. (2018). Feeding preference and biology of Tribolium castaneum Herbst (Coleoptera: Tenebrionidae) in different wheat varieties. J. Entomol. Zool. Stud..

[23] Lu H.H., Zhou J.C., Yan D., Zhao S.M., Xiong S.B. (2011). Effects of microwave radiation and conductive heating on Tribolium castaneum microstructure. Micron.

[24] Yao J., Chen C., Wu H., Chang J., Silver K., Campbell J.F., Arthur F.H., Zhu K.Y. (2019). Differential susceptibilities of two closely-related stored product pests, the red flour beetle (Tribolium castaneum) and the confused flour beetle (Tribolium confusum), to five selected insecticides. J. Stored Prod. Res..

[25] Deb M., Kumar D. (2020). Bioactivity and efficacy of essential oils extracted from Artemisia annua against Tribolium casteneum (Herbst. 1797) (Coleoptera: Tenebrionidae): An eco-friendly approach. Ecotoxicol. Environ. Saf..

[26] Iriti M., Faoro F. (2008). Oxidative stress, the paradigm of ozone toxicity in plants and animals. Water Air Soil Pollut..

[27] Kodrik D., Bednarova A., Zamanova M., Krishnan N. (2015). Hormonal regulation of response to oxidative stress in insects—An update. Int. J. Mol. Sci..

[28] Da Silva C.S., de Cássia Gonçalves de Lima R., Elekofehinti O.O., Ogunbolude Y., Duarte A.E., Rocha J.B.T., Alencar de Menezes I.R., Barros L.M., Tsopmo A., Lukong K.E. (2018). Caffeine-supplemented diet modulates oxidative stress markers and improves locomotor behavior in the lobster cockroach Nauphoeta cinerea. Chem. Biol. Interact..

[29] Holmstrup M., Sørensen J.G., Heckmann L.H., Slotsbo S., Hansen P., Hansen L.S. (2011). Effects of ozone on gene expression and lipid peroxidation in adults and larvae of the red flour beetle (Tribolium castaneum). J. Stored Prod. Res..

