# Supplementary material for: Assessing the Biological Safety of Atmospheric Cold Plasma Treated Wheat Using Cell and Insect Models
Source: Foods. 2020 Jul 8;9(7):898. doi: 10.3390/foods9070898 (PMC7404979; doi:10.3390/foods9070898)
Supplement: Supplementary file 1 [file foods-09-00898-s001.pdf]

Figure S1

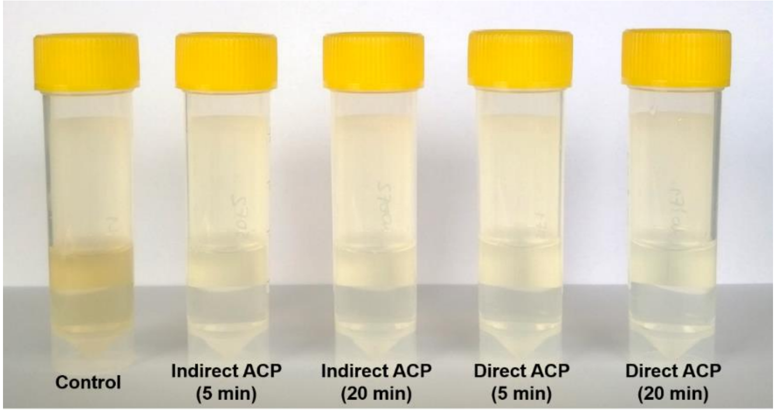

(a)

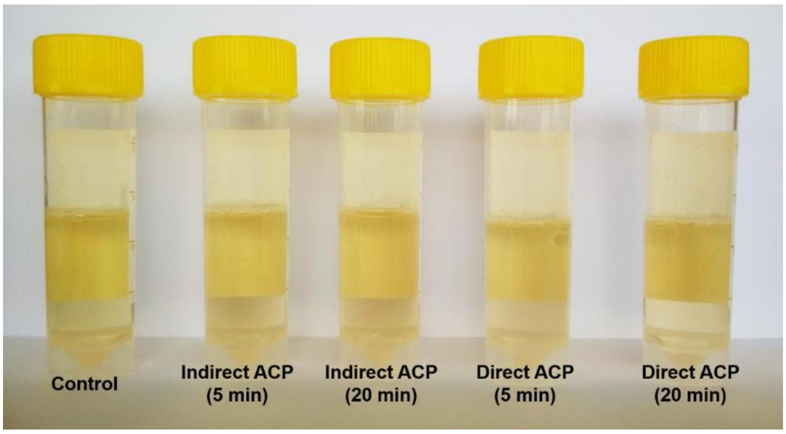

(b)

Figure S1. Change in the color of (a) WMM compared to (b) WGE after subjecting to ACP treatment.
